# The cardiovascular aspect of COVID-19

**DOI:** 10.1080/07853890.2020.1861644

**Published:** 2020-12-21

**Authors:** Joseph Adu-Amankwaah, Richard Mprah, Adebayo Oluwafemi Adekunle, Marie Louise Ndzie Noah, Gabriel Komla Adzika, Jeremiah Ong’achwa Machuki, Hong Sun

**Affiliations:** Department of Physiology, Xuzhou Medical University, Xuzhou, Jiangsu, China

**Keywords:** SARS-CoV-2, COVID-19, angiotensin-converting enzyme 2, acute respiratory distress syndrome, cardiovascular disease

## Abstract

The coronavirus disease-2019 (COVID-19), an infectious disease caused by Severe Acute Respiratory Syndrome Coronavirus 2(SARS-CoV-2), has hit the world very hard by affecting millions of people across countries hence posing a major health threat on a global scale. This novel virus is thought to enter and cause infection in its host through the attachment of its structural protein known as the S-glycoprotein to angiotensin-converting enzyme 2 (ACE2). Given the rapid spread of COVID-19 with its consequences globally, it is mandatory that health caregivers and researchers across all disciplines abreast themselves with the potential effects that this novel virus may have on their fields and the medical society at large. During the infection, the cardiovascular system is affected by unknown pathomechanistic processes, hence accounting for an increased prevalence of cardiovascular diseases (CVDs) among COVID-19 patients. As cardiovascular researchers, we are more concerned about the cardiovascular aspect of SARS-CoV-2/COVID-19. Hence, this concise review addresses these aspects where CVD as a risk factor of COVID-19, the prevalence of CVDs in COVID-19, and the potential cardiovascular disorders which may evolve owing to COVID-19 are discussed. A better understanding of these issues will be pivotal to improve cardiovascular health during this SARS-CoV-2/COVID-19 pandemic and beyond.

## 1. Introduction

Coronaviruses (CoVs) fall under the family Coronaviridae, which comprises enveloped viruses that have exceptionally big single-stranded RNA genomes extending from 26 to 32 kilobases in length [1]. CoVs have been detected in avians and several mammals, including a camel, bat, masked palm civets, and dogs. However, these viruses were previously regarded as pathogens that only cause mild diseases in immunocompromised patients till the emergence of coronaviruses causing SARS-CoV, MERS-CoV, and COVID-19 in 2002–2003, 2012 and 2019, respectively [1–5]. Currently, seven coronavirus species have been recognised to cause diseases in humans, namely, 229E, OC43, NL63, HKU1, SARS-CoV, MERS-CoV, and SARS-CoV-2 [1].

For the past two decades, the incidence of severe acute respiratory infections has been recognised as one of the major international health problems [6]. The severe acute respiratory syndrome (SARS-CoV) (2002) and Middle East respiratory syndrome (MERS-CoV) (2012) were estimated to have infected over 8422 and 1600 people respectively, and resulted over 916 and 574 deaths as well [7,8]. The current emergence of the 2019 novel coronavirus (2019-nCoV) infection which reportedly originated from Wuhan, Hubei, China in December 2019, has affected over 40 million people in over 200 countries/and territories after being declared a pandemic on 11 March 2020 by the World Health Organisation (WHO) [9].

Although COVID-19 is primarily a respiratory infection, it has adverse impact on the cardiovascular system (CVS) and resulting heart failures. Hence, from a cardiovascular standpoint, there are concerns as to whether patients with underlying cardiovascular conditions are at a higher risk of an aggravated COVID-19 infection. Also, as to whether COVID-19 infections induces cardiomyopathies that were not in existence prior to the infection. Previous studies have revealed a relationship between cardiovascular metabolic disorders, and the two prominent coronaviruses (SARS and MERS) [10,11]. Nonetheless, much needs to be discovered about the cardiac involvement of this novel coronavirus infection and its relationship with cardiovascular diseases (CVDs).

The purpose of this concise review is to discuss CVD as a risk factor of COVID-19, the prevalence of CVD in patients with COVID-19, and the potential cardiovascular disorders which may evolve owning to SARS-CoV-2 infection, as comprehending and addressing these issues will be crucial to improve outcomes during this critical period and beyond.

## 2. Search methodology

Literature review was done through systematical search for current finding mainly from Google Scholar and the National Centre for Biotechnology Information – PubMed database. The following defined words were used as search strategy to obtain relevant information: (SARS-CoV-2; Cardiovascular diseases; Epidemiology), (SARS-CoV-2; Cardiovascular diseases; Clinical features), (COVID-19; Cardiovascular diseases; Renin-Angiotensin System) and (COVID-19; Risk Factors; Mortality)

## 3. Epidemiology

Since its initial identification, the virus has extended to more than 180 countries across the world [12]. As of 7 April 2020, at 6:25:55pm, there have been a total of 1,407,123 confirmed cases, 80,759 deaths and 297,934 recoveries of COVID-19 reported globally, with the United States of America recording the highest confirmed cases (383,256), Italy with the most death cases (17,127) and china with the highest recoveries (77,410) [12]. In the initial phases of COVID-19, the epidemic elevated by two-folds in every 7.4 days, and the basic reproduction number was projected to be 2.2 [13]. Another study predicted the basic reproduction number as extending from 2.24 to 3.58 [14]. The infectivity of SARS-CoV-2 is higher compared to that of influenza, with a projected basic reproduction value of 2.28 [15]. Similarly, the mortality rate accompanying COVID-19 is also significantly higher than the most current WHO estimate of influenza death rate of below 0.1%. It may get to higher rates in patients with advanced age, those with underlying health conditions, and patients with inadequate intensive care support [5]. Although other zoonotic coronaviruses, including the 2002–2003 SARS-CoV epidemic and MERS-CoV, had higher case fatality rates of 9.6 and 34.4%, respectively [5,16], SARS-CoV-2 has caused many more deaths than both of these previous outbreaks combined [5,16,17]. Undefined and unpredictable disease definition has caused erraticism in reported case fatality rates for several reasons, including (1) the disease may present as asymptomatic or mildly symptomatic in a majority of patients [16], (2) poor testing abilities in most geographies, leading to common underdiagnosed, expressly in patients with mild illness, and (3) complications and death often antedate much later than contagion (normally between 2 and 3 weeks antedating infection) [5]. Notably, the assessment of SARS-CoV-2 infection may be further complicated owning to asymptomatic infection in a significant proportion of individuals (as much as 20%), which may significantly aid in the further spread of the infection [18].

## 4. Pathogeneses of SARS-CoV 2

SARS-CoV-2 is a beta coronavirus that encodes not less than 27 proteins, which comprises 15 non-structural proteins, 8 auxiliary proteins, and 4 structural proteins [19]. The 4 structural proteins include; the spike (S) glycoprotein, the nucleocapsid (N) protein, the envelope (E protein, and the membrane (M) protein [20]. The Spike glycoprotein (S), which is found on the surface of the virus, is responsible for the attachment to host receptor angiotensin-converting enzyme 2 (ACE2), therefore serving as a medium of cell entry [21]. Similarly, studies have exhibited that other coronaviruses can use the ACE2 protein to enter the cell [22,23]. ACE2 is a homolog of ACE and was discovered in 2000 [24]. It is a type I integral membrane protein that mediates many vital physiologic functions in the cardiovascular and immune systems of humans [5].In animal models, the expression of ACE2 in the heart plays a vital role; thus, ACE2 knockout mice develop severe left ventricular dysfunction [25]. ACE2 is active in most tissues and is widely dispersed in the heart, kidney, lung, and testis [24,26,27] and highly expressed in type 2 alveolar epithelial cells and endothelium, especially lung alveolar cells, offering the main entry site for the virus into human hosts [22,23]. Once ligand binding takes place, SARS-CoV-2 enters the cell through receptor-mediated endocytosis like human immunodeficiency virus (HIV) [28]. Thus, the S-glycoprotein on the outer surface of coronavirus binds to ACE2, leading to a dramatic structural rearrangement in the S-glycoprotein, thereby allowing proteolytic breakdown by host cell proteases (Transmembrane Serine Protease 2) eventually resulting in internalisation of the virus [29]. ACE2 also plays a role in lung protection, and hence viral binding to this receptor can affect the lung-protective pathway, contributing to viral pathogenicity [30]. Therefore, Viral S-glycoprotein, Transmembrane Serine Protease 2, and ACE2 inhibition can serve as potential targets of therapy and possibly vaccine development [29].

## 5. Clinical features of COVID-19

### 5.1. General clinical presentations

The clinical manifestation of COVID-19 varies widely owing to the fact that knowledge on its clinical features is still evolving [5,31,32]. SARS-CoV-2 infection can result in five different clinical outcomes: asymptomatically infected persons (1.2%); mild to medium cases (80.9%); severe cases (13.8%); critical cases (4.7%); and death (2.3%) among all reported cases [33]. A study done by the Chinese Centre for Disease Control and Prevention reported that among 72,314 patients with COVID-19 (16,186 suspected, 10,567 clinically-diagnosed and 44672 laboratory-confirmed), the clinical severity was documented as mild, severe and critical in 81.4, 13.9, and 4.7% of the patients respectively [16]. The clinical features exhibited by patients with mild COVID-19 included symptoms common to other viral infections such as; fever, cough, dyspnoea, anorexia, headache, nasal congestion, myalgia, sore throat, fatigue, and diarrhoea, which was accompanied by laboratory findings such as lymphopenia [34]. In severe COVID-19 cases, patients presented with pneumonia, acute respiratory distress syndrome (ARDS), with or without cardiogenic shock [5]. Signs and symptoms may manifest in 2–14 days of antedating exposure to an infected person [35]. Severe COVID-19 cases were also observed more in the aged populations as well as patients with pre-existing medical conditions [5,31,32,36]. Unlike influenza, COVID-19 exhibited a low prevalence of severe episodes in young children [37], thus among all the laboratory-confirmed episodes of COVID-19 in China, children were the least and seemed to be less vulnerable to this infection, perhaps due to stronger innate immunity, fewer underlying medical conditions, and differences in the maturation of viral receptors, and/or previous exposure to other coronavirus species [38]. Nevertheless, the moderate-to-severe disease has been reported in children also [39].

The imaging findings of COVID-19 also varies widely. Bilateral lung involvement was observed in more than 75% of patients [32,40], and multi-lobe involvement was also frequent among 71% of the patients [41]. Also, during chest computed tomography (CT) scans, ground-glass opacity (GGO) was the most frequent findings [32,42], where among 21 patients, GGO was seen in 86% during chest CT scans, and 29% exhibited consolidation [41]. Roughly one-third of the patients showed a peripheral distribution of GGO. On the other hand, no discrete nodules, cavitation, pleural effusion, or lymphadenopathy were seen in the chest images [41,42].

As yet, there are no standard preventative vaccines available for COVID-19. Similarly, there are no established guidelines specifically for treating COVID-19 patients, although several therapies are being busily reviewed [43].

### 5.2. Cardiovascular clinical presentations

SARS-CoV-2 attacks the respiratory system, but has adverse effects on the CVS [44]. As such, patients with COVID-19 manifest several clinical characteristics arising from cardiovascular conditions such as myocardial injury, myocarditis, acute coronary syndrome (ACS), acute myocardial infarction (AMI), cardiac arrhythmia, cardiac arrest, venous thromboembolic disease and heart failure [45]. Laboratory findings among COVID-19 patients in Wuhan revealed an increase in high-sensitivity cardiac troponin I (hs-cTnI), creatine kinase (CK)-MB [31], C-reactive protein and N-terminal pro-brain natriuretic peptide (NT-proBNP) levels [44]. Also, an electrocardiogram (ECG) from a COVID-19 patient showed a minimal diffuse ST-segment elevation which was more perceptible in the inferior and lateral leads and ST-segment depression with T-wave inversion in lead V1and aVR [44].

## 6. The impact of COVID-19 on the cardiovascular system

**Figure 1.**
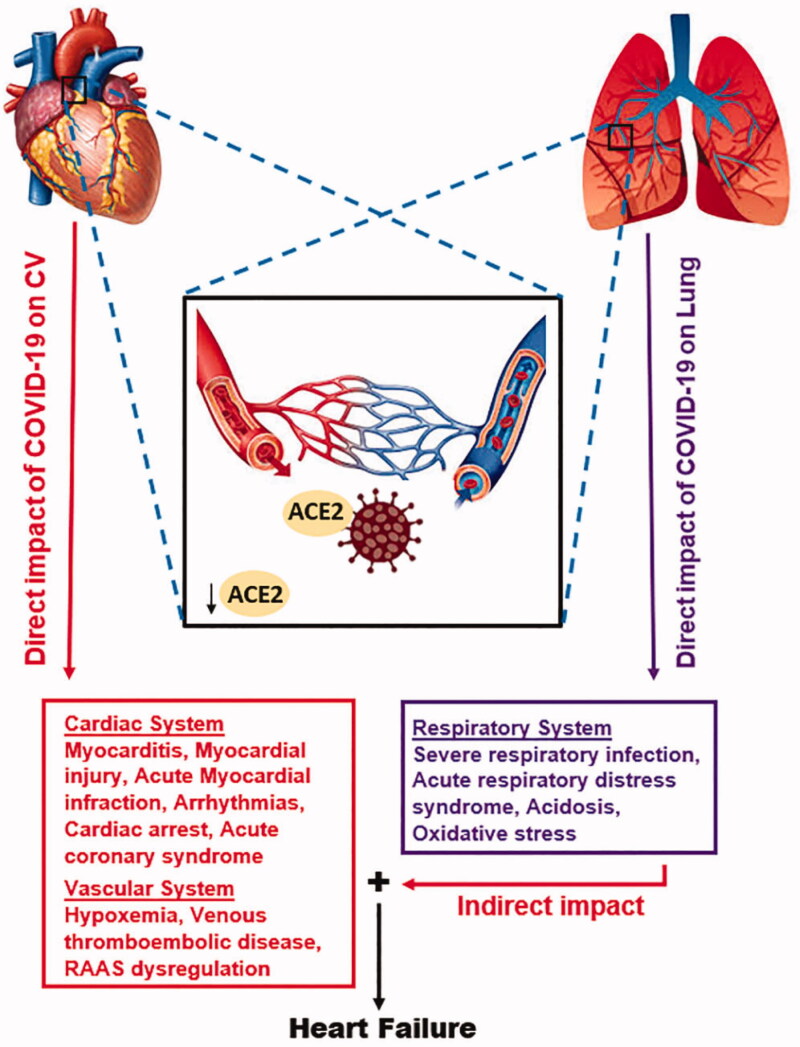
Schematic of the probable mechanisms for the direct and indirect impact of COVID-19 on CVS. The direct effect is initiated in the CVS while, the indirect effect begins in the lung when the SARS-CoV-2 Spike protein attaches to ACE2. In both instances, ACE2 are internalised, thereby, disrupting the exertion of its protective effects. Directly, ACE2 downregulation along with other pre-existing CV complications exacerbates CV dysfunction. Indirectly, ACE2 downregulation in the lungs induces respirational dysfunctions which affects CV function by induction of hypoxaemia, ischaemia, myocardial and other CV complications. The effect of SARS-CoV-2 on the respiratory system, coupled with the likely synergy (**+**) of its direct and indirect impacts on the CVS might be resulting in the heart failures occurring in COVID-19 patients.

SARS-CoV infections tend to downregulate ACE2, which might contribute to myocardial dysfunction hence affecting the CVS at large [46]. However, whether SARS-CoV-2 directly affects the CVS by targeting ACE2-expressing cells remains to be clarified [47]. Another theory may involve an indirect effect of the immune response to SARS-CoV-2 on the heart along with the blood vessels [47] (see Figure 1*).* A 12- year follow-up study of 25 patients who recuperated from SARS-CoV infection revealed that 68% developed hyperlipidaemia, 44% developed CVS disorders, and 60% experienced abnormalities of glucose metabolism [48,49]. Additionally, metabolomics analysis reported that the deregulation of lipid metabolism occurred in patients having a history of SARS-CoV infection. In these patients, the serum levels of lysophosphatidylcholine, free fatty acids, phosphatidylglycerol, and lysophosphatidylethanolamine were significantly elevated compared with those without a history of SARS-CoV infection [48,49]. Nevertheless, the underlying processes by which SARS-CoV infection causes lipid and glucose metabolic disorders remains unclear. In a study of 75 hospitalised SARS patients, acute myocardial infarction (AMI) was the cause of mortality in 2 out of 5 severe cases [50]. Given that COVID-19 has a comparable structure and pathogeneses of SARS-CoV, then this novel virus can also cause chronic damage to the cardiovascular system; hence cardiovascular protection during treatment for COVID-19 should not be disregarded [49].

## 7. CVD as a risk factor of severe COVID-19

According to previous studies on SARS-CoV, the existence of underlying medical conditions such as cardiac disorders and diabetes increased the risk of mortality among patients. Thus, cardiac disorders and diabetes elevated the risk of death by twice as much as other risk factors [51]. Similarly, a meta-analysis exhibited that MERS-CoV infection was more probable to happen in patients with underlying CVD [6]. Currently, patients with pre-existing CVDs are among individuals with the highest risk of developing severe COVID-19, which may result in worse clinical outcomes [29]. Hence, individuals with CVD account for a large proportion of deaths from COVID-19 [49]. According to the mortality data given by the National Health Commission of China (NHC), 17% and 35% of individuals with COVID-19 presented a history of coronary heart disease and hypertension respectively [49]. Li et al. [52] revealed that the presence of cardio-cerebrovascular disease, diabetes, and hypertension heightened the risk of experiencing severe COVID-19 by 3-fold, 2-fold, and 2-fold, respectively. Additionally, a much larger study by the Chinese Centre for Disease Control and Prevention describing clinical outcomes in 44,672 confirmed episodes of COVID-19 disclosed that the case fatality rate (CFR) was 2.3% in the whole cohort but significantly higher (6, 7.3, and 10.5%) in individuals with hypertension, diabetes and CVDs respectively [16]. Several smaller cohort studies have also given similar reports proposing higher risk for an adverse episode in COVID-19 patients with underlying CVD [5,31,32]. Cardiac injury (characterised by increased troponin levels), myocarditis, as well as ARDS has been reported as strong and autonomous risk factors linked with mortality in COVID-19 patients [35]. Based on Pneumonitis Diagnosis and Treatment Program for New Coronavirus Infection, aged (>60 years) with pre-existing medical conditions are more likely to be infected with COVID-19, particularly those with hypertension, coronary heart disease or diabetes [49]. Thus, advanced age, male sex, and the existence of underlying medical conditions are noted to be the main risk factors for COVID-19 mortality [37]. Nevertheless, so far, the underlying processes of these associations remain unclear. Three theories have been proposed associated with ACE2 expression that could enlighten the relationship between pre-existing CVD and severe COVID-19 with worse outcomes; these theories include; 1) Population disparities in ACE2 expression or function can concurrently heighten the risk of experiencing CVD and increase the likelihood of severe SARS-CoV-2 infection. 2) CVD itself or the therapeutic renin–angiotensin system (RAS) blockade used in CVD management can enhance ACE2 expression, increasing available binding sites within organs such as the lung and heart for the evolution of SARS-CoV-2 infection. 3) The SARS-CoV-2 infection might downregulate ACE2 function, probably to a greater degree in individuals with underlying CVDs [53]. Similarly, CVD is more prevalent in those with advancing age, and hence a functionally impaired immune system could also contribute to the heightened risk of severe COVID-19 [54].

## 8. Prevalence of CVD in patients with COVID-19

Although, several studies have proposed an association between CVDs and severe COVID-19, the lack of national surveillance, widespread testing and standardised data collection has complicated efforts to perfectly estimate the prevalence of CVDs in COVID-19 patients [5] However, a present systematic analysis which summarised data from six studies among 1,527 COVID-19 patients in China, revealed that the proportion of patients with hypertension, cardio-cerebrovascular disease and diabetes were 17.1, 16.4, and 9.7%, respectively [52]. Though the prevalence of diabetes and hypertension in this cohort was comparable to that in the overall Chinese population, the prevalence of cardio-cerebrovascular disease was significantly elevated [55]. In a small retrospective study among 150 laboratory-confirmed COVID-19 patients, which analysed the factors associated with mortality indicated that CVD was commonly observed in patients who died (13 of 68) than patients who recovered (0 of 82) [56]. Additionally, among the confirmed cases of COVID-19 reported by the National Health Commission of China (NHC), some of the patients initially went for consultation due to CVD manifestation. Thus, the patients went with chest tightness and heart palpitations instead of respiratory presentations, such as cough and fever, but were diagnosed with COVID-19 later. According to NHC, among the individuals who died from COVID-19, 11.8% of them without pre-existing CVD had significant heart damage and cardiac arrest, with increased levels of cardiac troponin I during hospitalisation [49]. Hence, in patients with COVID-19, the prevalence of cardiovascular manifestations is high due to the systemic inflammatory response and immune system disorders during disease progression [49]. Cardiac troponin I and natriuretic peptides levels are significantly elevated in patients with severe COVID-19 than those with milder forms of the disease [57]. This may be similar to what is seen in many patients with acute respiratory illnesses, or it may show myocardial injury since the virus binds to ACE2 receptors, which are widely expressed in the pericytes of the heart [29,46,58]. The prevalence of cardiovascular, metabolic diseases among COVID-19 patients is shown in Table 1.

**Table 1.** The prevalence of cardiovascular, metabolic diseases in COVID-19 patients.

Number of patients (*N*)	Prevalence of cardiovascular metabolic diseases (%)	References
138	68.1	Wang et al. **[**32**]**
41	62.0	Huang et al. [31]
1099	39.9	Guan et al. [67]
99	75.0	Chen et al. [40]
137	36.5	Liu et al. [68]
150	0.1	Ruan et al. [56]

## 9. Cardiovascular disorders associated with COVID-19

### 9.1. Myocardial injury and myocarditis

Myocardial injury, which is characterised by an elevated level of troponin, can occur antedating myocardial ischaemia or non-ischemic myocardial processes such as myocarditis [5,59]. According to previous studies, the MERS-CoV can cause heart failure and acute myocarditis in infected patients [49]. Since SARS-CoV-2 and MERS-CoV have akin pathogenicity [49], myocardial injury can also occur in individuals with COVID-19 due to myocarditis and hypoxia antedating severe respiratory infection and ARDS. SARS-CoV-2 tends to affect the myocardium and cause myocarditis [60]. Findings from autopsy reports propose an infiltration of myocardium through interstitial mononuclear inflammatory cells, which consist of macrophages and, to a lesser extent, CD4+ T cells [46,60]. Similarly, cases of severe myocarditis with decreased systolic function have been seen in patients with COVID-19 [35]. Increased levels of biomarkers, such as serum troponin and natriuretic peptides, have been reported among several COVID-19 patients [56,59]. In a meta-analysis of four studies among 341 patients, it was observed that patients with severe COVID-19 had significantly higher troponin I level than those with mild disease (25.6, 95% CI 6.8-44.5) [57]. Reports have also revealed that acute cardiac injury, which includes elevation of cardiac biomarkers greater than 99th percentile of the upper reference limit in the presence of electrocardiographic and echocardiographic disorders, is notably common COVID-19 patients and is linked with more severe disease and worse outcomes. Undeniably, the mortality risk accompanying acute cardiac injury in COVID-19 patients was more significant in elderly patients with diabetes, chronic pulmonary disease, or prior history of CVD [46,61]. Several cohort studies among hospitalised patients in China stated that myocardial injury occurred in 7–17% of COVID-19 patients [31,32,36], which was significantly more frequent in those admitted to the ICU (22.2 vs. 2.0%, *p* < 0.001) and among patients who died (59 vs. 1%, *p* < 0.0001) [23,36]. As yet, nevertheless, no data is exhibiting the presence of SARS-CoV-2 within myocardial tissue [46]. The underlying processes of acute myocardial injury resulting from COVID-19 infection may be associated with ACE2, since it is highly distributed not only in the lungs but also in the CVS and, hence, ACE2-related signalling pathways may play a role in heart injury [49]. Other suggested pathomechanisms of myocardial injury include a cytokine storm initiated by an imbalanced response of type 1 and type 2 T-helper cells [49] and respiratory dysfunction as well as hypoxaemia caused by COVID-19, leading to damage of myocardial cells [49].

### 9.2. Acute coronary syndrome (ACS)

ACS, which involves acute myocardial infarction (AMI) may occur in COVID-19 patients; however, the incidence of such events is not clear [46]. Hypothetically, the risk for ACS in affected patients may be elevated owing to an increased thrombotic tendency, as corroborated by significantly elevated levels of D-dimers [46]. Additionally, clinical studies on prior epidemics support these observations by showing a strong association between viral respiratory infections and ACS (incidence ratio for ACS within 7 days of infection: 2.8–10.1) [46,62]. Although data on the scope of ACS in COVID-19 are limited, yet, these disorders contributed to in-hospital death in the SARS epidemic [46]. Lastly, the symptoms of infection and the elevated incidence of non-ischemic cardiac injury may masquerade as ACS (consisting of electrocardiographic anomalies, high troponin levels, and chest pain); hence a high index of suspicion for alternative diagnosis is necessary [46].

### 9.3. Cardiac arrhythmia and cardiac arrest

Cardiac arrhythmias and cardiac arrest, ranging from tachycardia and bradycardia to asystole are cardiovascular presentations, which are also often reported in COVID-19 patients [5,46]. Arrhythmias in COVID-19 patients can arise secondary to hypoxaemia, metabolic disorders, systemic inflammation, or myocarditis [46]. A cohort study among 137 COVID-19 patients revealed that 7.3% presented with non-specific heart palpitations [26]. In another study among 138 hospitalised COVID-19 patients in China, cardiac arrhythmia was observed in 16.7% and was more prevalent in ICU patients than non-ICU patients (44.4 vs. 6.9%) [32]. In China, a projected 11.8% of patients who died from COVID-19 manifested considerable heart damage with cardiac arrest in the course of hospitalisation, without having any pre-existing cardiovascular diseases [49]. However, specifics about the forms of arrhythmias that are seen in these patients are yet to be published. Elevated prevalence of arrhythmias may be a result of hypoxia, metabolic disarray, neurohormonal or inflammation antedating viral infection in individuals with or without pre-existing CVD. Nevertheless, new onset of malignant tachyarrhythmia in the presence of elevated troponin should raise the alarm for underlying myocarditis [5,56].

### 9.4. Cardiomyopathy and heart failure

According to Zhou et al., 23.0% of COVID-19 patients came with heart failure [59]. Also, Mohammed et al. [63] reported takotsubo cardiomyopathy as a complication of a COVID-19 patient having a history of non-ischemic cardiomyopathy (NICM) with left ventricular ejection fraction (LVEF) of 15%. A cohort study done by Driggin et al. [5) observed that heart failure was often seen in COVID-19 patients than acute kidney injury and was more frequent in patients who died than those who did survive during hospitalisation (51.9 vs. 11.7%). Besides, among causes of death in a Wuhan owing to COVID-19, myocardial damage and heart failure accounted for 40%, either entirely or in combination with respiratory failure [46,61,64]. A current study concluded that patients with basic heart failure disease exhibited higher expression of ACE2, and may have elevated risk of a heart attack, which can progress to severe conditions after infection [58]. Nevertheless, there are inadequate data to demonstrate whether myocarditis in COVID-19 frequently causes heart failure with reduced ejection fraction (HFrEF) or preserved ejection fraction (HFpEF) [46]. Additionally, it is still not elucidated whether heart failure frequently occurs owing to aggravation of pre-existing left ventricular dysfunction or newly occurring cardiomyopathy (either due to lifestyle or chronic catecholamine stress), hence needs to be clarified [5,65]. Associated pulmonary hypertension and right heart failure should also be explored, specifically in the setting of severe ARDS and parenchymal lung disease [5].

### 9.5. Venous thromboembolic disease

Patients with COVID-19 are probably at heightened risk of experiencing venous thromboembolism (VTE). Although there are limited published case series so far, reports of abnormal coagulation parameters have been described in hospitalised patients with severe COVID-19 [5,66]. A multicenter retrospective study carried out in China disclosed that increased levels of D-dimer (>1 g/L) were significantly associated with in-hospital mortality, even after multivariable adjustment (OR 18.4 95% CI 2.6–128.6, *p* = 0.003) [59]. Similarly, another study contrasting COVID-19 survivors to non-survivors, observed that higher fibrin degradation products (FDP) and D-dimer levels were significantly associated with non-survivors and 71.4% of them suffered disseminated intravascular coagulation (DIC) during their disease progression [66]. The thromboembolic disease should be regarded in seriously ill COVID-19 patients who exhibit worse clinical outcomes, as evidenced by hypoxia or hemodynamic instability[5].

## 10. Conclusions and future directions

The COVID-19 pandemic has hit the world very hard by affecting thousands of people across countries posing a major health threat on a global scale. This novel virus is thought to enter and cause infection in its host through the attachment of its structural protein known as the S-glycoprotein to ACE2, which are highly expressed on the host cells. Thus, causing damage to the CVS through unknown pathomechanistic processes and therefore accounting for the increasing prevalence of CVDs among COVID-19 patients. Furthermore, patients with underlying CVDs are at a higher risk of experiencing severe COVID-19 with adverse outcomes. Hence, specific heed should be paid to cardiovascular protection during the management and treatment of COVID-19 patients.

Although it is clear that COVID-19 has adverse outcomes on the cardiovascular system, what remains unknown is the pathomechanistic process in which it occurs. Therefore, several studies are needed to clarify these underlying mechanisms. Considering the high expression of ACE2 in the cardiovascular system, especially on the myocardium, it can be proposed that ACE2-related signalling pathways owning to COVID-19 might play a chief role in damaging the cardiovascular system. Additionally, a better apprehension of the relationship between the antihypertensive agent used in CVD management and the COVID-19 prognosis will have vital implications for individuals with both COVID-19 and pre-existing CVD.
